# Factors correlated with personal growth initiative among college students: A meta-analysis

**DOI:** 10.1016/j.heliyon.2024.e28518

**Published:** 2024-03-25

**Authors:** Zun Jiao, You Chen, Chunwei Lyu

**Affiliations:** a Department of Literature, Qingdao Film Academy, Qingdao, China; b School of Housing, Building & Planning, Universiti Sains Malaysia, Penang, Malaysia; c School of Educational Studies, Universiti Sains Malaysia, Penang, Malaysia

**Keywords:** Personal growth initiative, College students, Correlated factors, Meta-analysis

## Abstract

In higher education, Personal growth initiative (PGI) has been the focus of attention, personal growth initiative is a fundamental mechanism for individual advancement, equipping college students with the resilience to navigate obstacles and bolstering self-enhancement. The present study comprehensively synthesizes existing research on the factors correlated with personal growth initiative among collegiate populations, aims to identify all correlated factors of college students' personal growth initiative, and the level of correlation. A systematic search was conducted through Scopus, Web of Science, PubMed, JSTOR, PsycINFO, ScienceDirect, and Wiley Online Library, as well as manually search in Google Schalor, spanning to collate research on college students' personal growth initiative. Quantitative synthesis was performed using STATA 17, while sensitivity was tested using a change effect model approach and publication bias was assessed employing Egger's test. After applying the Bonferroni correction, this study found that 18 factors were significantly positively correlated with college students' personal growth initiative, including 4 high-level correlated factors, 10 medium-level correlated factors, and 4 low-level correlated factors, as well as 4 factors were significantly negatively correlated, including 1 medium-level correlated factor and 3 low-level correlated factors. These findings offer valuable insights into personal growth initiative among college students, and the reference for educators and institutional leaders aiming to foster personal growth initiative for college student self-development.

## 1 Introduction

The period of college students is a critical stage full of challenges and growth. As college students transition from adolescence to adulthood [1], they grapple with a multitude of pressures from various aspects [2,3], such as academics, careers, and interpersonal relationships. At this stage, the Personal Growth Initiative (PGI) plays an important role. Personal growth initiative is seen as a valuable resource that empowers college students to enact positive change and foster personal growth [4]. Comprising components Readiness for Change, Planfulness, Using Resources, and Intentional Behavior [5], personal growth initiative is not only a kind of will, but also a kind of action, embodies a proactively pursue to self-improvement and self-development. Research suggests that people with strong personal growth initiative are more adept at coping with life's challenges and are better equipped to navigate transitions positively [5,6]. Therefore, with good personal growth initiative is very important for college students as they navigate the path toward independent adulthood [7].

In recent years, an increasing number of studies have emerged concerning college students' personal growth initiatives. There have been many factors that have been considered by scholars as important factors correlated with college students' personal growth initiative. For instance, self-efficacy is theorized to be the basis of the personal growth initiative [8], studies also supporting a significant positive correlation between self-efficacy and personal growth initiative among college students [9,10]. Additionally, research has indicated significant correlations between personal growth initiatives and factors well-being [11], self-esteem [12], hope [13] among college students. However, the majority of current research on college students' personal growth initiative comprises single cross-sectional or survey studies [14], given the large number of factors correlated to the personal growth initiative among college students, individual cross-sectional or survey studies are limited in their ability to cover all correlated factors comprehensively. Additionally, various limitations inherent in single studies have resulted in inconsistent findings regarding the factors correlated with personal growth initiatives among college students. For example, while a 2007 study found a significantly and negatively correlation between distress and college students’ personal growth initiatives [15], but a 2012 study reported that the correlation was not significant [11].

Therefore, existing research on the factors correlated with personal growth initiative among college students are scattered and inconsistent, and there is a noticeable lack of comprehensive and systematic evidence-based research with large sample sizes. To address this gap, this study conducted a meta-analysis to identified all the factors correlated with college students' personal growth initiative and their level of correlation involved in current-day research, aim to enhance understanding of college students' personal growth initiative and provide constructive reference for the cultivation of college students’ personal growth initiative.

## 2 Literature review

### 2.1 Personal growth initiative among college students

In recent years, nurturing personal growth initiatives among college students has emerged as a significant concern within the realm of higher education [16,17]. In this era marked by diversity and progress, fostering personal growth initiatives among college students is not only essential for keeping pace with the rapidly evolving knowledge landscape, but also aims to empower them to navigate their life paths with greater determination and confidence [18]. Consequently, the influence of personal growth initiatives on college students extends beyond academics to encompass social and career development [19], exerting a positive impact on shaping their future.

Meanwhile, in accordance with Erikson's eight-stage theory of individual personality development, college students are in early adulthood stage [1], this is an important period of transition, with a variety of changes and developmental tasks to be addressed [2,3]. Failure to address these tasks adequately can result in negative emotional, behavioral, and cognitive consequences [20]. Numerous scholars have contended that good personal growth initiatives are necessary to respond positively to life's challenges and adapt positively to change [5,6]. Therefore, personal growth initiatives are important for college students facing the challenges of transitioning from adolescence to independent adulthood [7].

### 2.2 Existing research on personal growth initiative of college students

Most studies on personal growth initiatives are cross-sectional empirical studies aimed at investigating the current level of personal growth initiatives and the correlated factors within specific populations [14]. Consequently, scholars have developed various versions of the Personal Growth Initiative Scale to assess individuals' personal growth initiative levels based on its conceptual framework and characteristics, conducting research accordingly. Among the most widely used scales in studies involving college student populations are the Personal Growth Initiative Scale (PGIS) [21] and the Personal Growth Initiative Scale-II (PGIS-II) [5]. Existing research on the factors correlated with personal growth initiatives among college students indicates that individual psycho-protective factors such as self-esteem and self-efficacy have been found to correlated with college students’ personal growth initiatives [22,23] and have study also found that personal growth initiatives among college students are closely correlated with mental health [24].

However, current empirical studies generally suffer from insufficient coverage, inconsistent findings, and a lack of large samples, making it impossible to identify all the factors that correlated with college students' personal growth initiatives, much less the specific level of correlation. The most effective way to address the issues above is through comprehensive and systematic evidence-based research [25]. However, there are only two existing research evidence review articles on personal growth initiatives, one of which is a systematic review [14] that only introduces factors correlated to personal growth initiatives at the theoretical level and does not identify the specific level of correlation. The other one is a meta-analysis [24] that only explores the relationship between personal growth initiatives and mental health, while the extent of the correlation between personal growth initiatives and mental health was identified, it was limited to two factors (distress and well-being), with the correlated factors having very limited coverage. Obviously, both two studies have their own limitations and the above issues have not been well addressed, as well as the populations in these two studies are not college students. Therefore, there is necessary for a comprehensive evidence-based study to identify all correlated factors of college students' personal growth initiatives and their level of correlation, in order to better understand college students’ personal growth initiatives.

### 2.3 The present study

To fill the gap in evidence-based research on personal growth initiatives among college students, this study presents a meta-analysis of personal growth initiatives among college students. The first research objective of the present study is to determine all the factors correlated with college students' personal growth initiatives in current-day research, and the second research objective is to identify the level of correlation between each correlation and college students’ personal growth initiative.

## 3 Method

The study adhered to the Cochrane guidance for conducting systematic reviews [26]. The PRISMA checklist [27] was followed to reporting. The study protocol outlining was published in PROSPERO (PROSPERO ID: CRD42023432412).

### 3.1 Search strategy

This study searched Seven databases (Scopus, Web of Science, Pubmed, JSTOR, PsycINFO, ScienceDirect, and Wiley Online Library) and manually to find articles that articles with (“Personal Growth Initiative” OR “PGI”) AND (“college student” OR “university student” OR “undergraduate”) in the all filed, the search years are Start of the database build to February 1, 2024. Since this study focuses only on factors correlated with personal growth initiatives among college students, a direct search in all field of all databases was conducted. A search strategy was developed in the Web of Science database and applied to the other electronic databases.

### 3.2 Inclusion and exclusion criteria

For studies to be conducted in any country but must be published in English, published in other languages will be excluded. Those studies published after February 1, 2024 will be excluded. Must have full text, the sample or participants must be college students and studies with other samples or participants will be excluded. Studies must report correlations between Personal Growth Initiative and correlated factors among college students, with outcomes of Pearson's correlation coefficient (r) or Spearman's correlation coefficient (rs), studies with other outcomes will be excluded. The reliability and validity of all the measurement tools are also reported, if not, will be excluded. Studies rated as high risk of bias will be excluded.

### 3.3 Screen studies and data extraction

After entering all the retrieved studies into the Endnote Library and removing duplicates, each study was checked based on its title, abstract and full text. Firstly, some articles were excluded because of their titles. Secondly, the article abstracts are then evaluated and those that do not fit are excluded. Thirdly, a full-text review and assessment of the remaining articles. For the final included articles, Data extraction was carried out independently by both authors using a designed data extraction form, including authors, year of publication, study site, sample size, correlated factors and their outcomes, and final results were cross-checked.

### 3.4 Quality assessment

The quality of the reviews was identified using the Joanna Briggs Institute (JBI) analysis cross-sectional study checklist [28]. Two authors independently assessed the included studies and if there were disagreements, the third author was invited to negotiate a resolution. If the ≥4 categories on the JBI tool were rated as low quality [29], the study was excluded as this would be seen as a significant problem in the study design and implementation process.

### 3.5 Data analysis

Firstly, this study will statistically count the frequency of all relevant factors extracted from the included articles, a word cloud will display the current research hotspots on personal growth initiative's correlated factors among college students. Secondly, the correlation coefficients between college student's Personal Growth Initiative and correlated factors were transformed as follows: 1) Fisher’ Z = 0.5 × ln [(1 + r)/(1 - r)]; 2) Vz = 1/(n - 3); 3) SE = Vz. Then converted data was analyzed using STATA 17 software, and the χ^2^ test was used to determine whether there was heterogeneity between studies. If P > 0.1 and I^2^ < 50%, heterogeneity between studies was considered acceptable and a fixed-effects model was chosen; if P < 0.1 and I^2^ > 50%, heterogeneity between studies was considered to exist and a random-effects model was chosen [30]. Sensitivity was tested using a change analysis model approach. Egger's test was used to assess publication bias, if p < 0.05 that have publication bias [31]. Finally, Summary r was used to synthetically evaluate the correlation between college students' Personal Growth Initiative and correlated factors: Summary r = (e2z - 1)/(e2z + 1), using P-values to assess whether the correlation was significant or not, and to avoid Type I error, corrected using the Bonferroni method, with P < 0.05/number of analysis considered statistically significant [32]. Pearson correlation coefficient level standard: |r| ≤ 0.3 low correlation; 0.3 < |r| < 0.5 medium correlation; |r| ≥ 0.5 high correlation [33].

## 4 Results

### 4.1 Search results

**Fig. 1:**
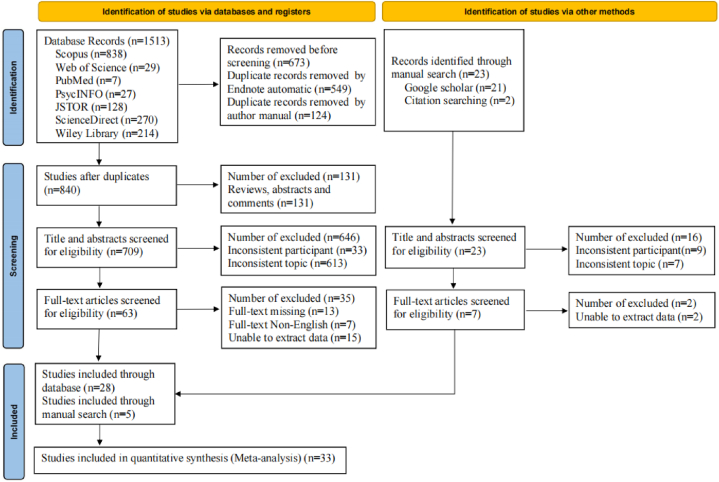
Flow diagram for included studies.

This study retrieved 1513 records from the database search and added 23 records from the manual search. For databases records, after removing the duplicate articles using Endnote, 840 studies were retained. Of these studies, 131 were excluded from the initial screening because these article types were not accepted for this review. For the remaining 709 studies, titles and abstracts are accessed and screened. Of these studies, 646 were excluded because of studies where the sample was not college students or did not meet the requirements topic. Of the remaining 63 studies, 13 studies was excluded because the full text was missing, 7 studies was excluded because the full text was non-English, 15 studies were excluded because they did not report Pearson's correlation coefficient or Spearman's correlation coefficient, our attempts to contact the authors at their email addresses to obtain the study data were not returned, thus 28 studies included in this meta-analysis from databases. As well as using the same steps to screen the manually searched studies, 5 studies matches the criteria. Finally, this study included 28 studies from database search and 5 studies from manual search for meta-analysis (Fig. 1).

### 4.2 Description of studies

The characteristics of the included studies are shown in Table 1. All studies used cross-sectional surveys design, a total of two measures were applied in 33 studies to measure personal growth initiative among college students, separate different language versions of the Personal Growth Initiative Scale (PGIS) [21] and Personal Growth Initiative Scale-II (PGIS-II) [5], both had good reliability and validity (Different language versions of the same scale are considered the same). The correlated factors involved in all 33 studies were also measured reliably and reported Pearson's correlation coefficients with college students' personal growth initiatives.

**Table 1:** Characteristic of included studies.

Author	Year	Location	Sample details	PGI measure	Correlated Factors
Neff et al. [34]	2007	The USA	177 College students Mean age: 20.2	PGIS	Self-Compassion; Subjective Happiness; Optimism; Positive Affect; Negative Affect
Hardin et al. [15]	2007	The USA	134 College students Mean age: 19.52	PGIS	Psychological Distress; Positive Affect; Negative Affect; Fear of Negative Evaluation
Ogunyemi & Mabekoje [35]	2007	Nigeria	425 College students Mean age: 22.40	PGIS	Risk-Taking; Self-Efficacy; Mental Heath
Shorey et al. [36]	2007	The USA	378 College students Mean age: 18.90	PGIS	Psychological Distress; Psychological Well-Being; Hope; Optimism
Oluyinka et al. [37]	2011	Nigeria	452 College students Mean age: 23.34	PGIS	Mindfulness
Weigold & Robitschek [4]	2011	The USA	478 College students Mean age: 20.64	PGIS	Problem-Solving Confidence; Personal Control; Anxiety
Ayub & Iqbal [11]	2012	Pakistan	150 College students Mean age: 19.45	PGIS	Well-Being; Psychological Distress
Malik et al. [12]	2013	Pakistan	150 College students Mean age: 19.55	PGIS-II	Self-Esteem; Academic Achievement
Yakunina et al. [38]	2013	The USA	386 College students Mean age: 24.00	PGIS-II	Acculturative stress; Psychological Adjustment
Loo et al. [13]	2014	China	801 College students Mean age: 25.36	PGIS	Mindfulness; Hope
Luyckx & Robitschek [39]	2014	Belgium	551 College students Mean age: 19.26	PGIS-II	Self-Esteem; Depression
Çelik [10]	2015	Turkey	237 College students Mean age: 20.13	PCIS	Student Academic Support; Self-Efficacy

Author	Year	Location	Sample details	PGI measure	Correlated Factors
Beri & Jain [9]	2016	India	480 College students Mean age: Non-report	PGIS-II	Emotional Self-Efficacy; General Well-Being
Borowa et al. [40]	2016	The USA	136 College students Mean age: 30.92	PGIS-II	Social Support
Yang & Chang [41]	2016	The USA	227 College students Mean age: 19.7	PGIS-II	Hope; Life Satisfaction; Optimism; Depression; Anxiety
Batool et al. [42]	2017	Pakistan	300 College students Mean age: Non-report	PGIS	Self-Efficacy
Çankaya et al. [8]	2017	The USA	188 College students Mean age: 27.74	PGIS-II	Self-Efficacy
Shigemoto et al. [43]	2017	The USA	292 College students Mean age: 19.91	PGIS-II	Depression; Posttraumatic Stress
Hirata & Kamakura [44]	2018	Japan	329 College students Mean age: 20.50	PGIS-II	Self-Esteem
Kugbey et al. [45]	2018	Ghana	260 College students Mean age: 21.72	PGIS-II	Emotional Intelligence; Subjective Happiness
Aranha et al. [22]	2019	India	120 College students Mean age: Non-report	PGIS	Self-Compassion; Self-Esteem
Mason [46]	2019	South Africa	235 College students Mean age: 20.38	PGIS	Subjective Happiness; Well-Being
Arikkatt & Mohanan [47]	2020	Thailand	346 College students Mean age: Non-report	PGIS-II	Self-Compassion; Well-Being
Noor et al. [48]	2020	Malaysia	225 College students Mean age: 20.92	PGIS-II	Life Satisfaction

Author	Year	Location	Sample details	PGI measure	Correlated Factors
Cai & Lian [49]	2021	China	1912 College students Mean age: 19.32	PGIS-II	Social Support; Self-Efficacy; Sense of Purpose
Chowdhury et al. [50]	2021	India	156 College students Mean age: Non-report	PGIS	Self-Esteem
Hassan et al. [51]	2021	Pakistan	400 College students Mean age: Non-report	PGIS-II	Emotional Intelligence; Subjective Happiness
Masood & Arshad [52]	2021	Pakistan	198 College students Mean age: 24.00	PGIS-II	Well-Being; Family Functioning
Soylu et al. [53]	2021	Turkey	2255 College students Mean age: 21.32	PGIS-II	Optimism
Weigold et al. [18]	2021	The USA	818 College students Mean age: 20.13	PGIS-II	Well-Being; Self-Determination
Chen & Guo [54]	2022	China	783 College students Mean age: 20.34	PGIS-II	Smartphone Addiction; Personal Control
Green & Yıldırım [55]	2022	Pakistan	461 College students Mean age: 24.66	PGIS	Life Satisfaction
Rahim et al. [56]	2024	Pakistan	300 College students Mean age: 20.29	PGIS-II	Mindfulness

Notes: PGIS=Personal Growth Initiative Scale; PGIS-II=Personal Growth Initiative Scale-II.

**Fig. 2:**
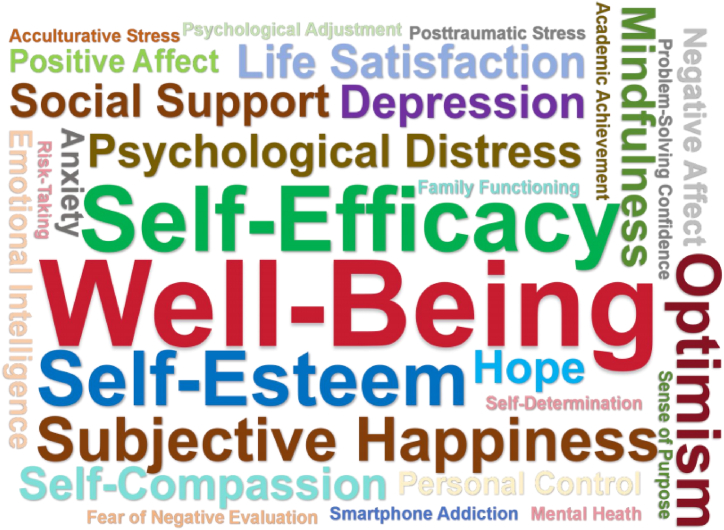
Word could for correlated factors.

From the 33 articles, 29 different correlated factors were extracted. Well-being was the most frequent, with 7 studies involving well-being; followed by self-efficacy, with 6 studies involved. Meanwhile, smartphone addiction, fear of negative evaluation, academic achievement, and other 10 correlated factors were each involved in only one study. Based on the frequency of each correlated factor, the generated word cloud is shown in Fig. 2.

### 4.3 Quality assessment results

The quality assessment results were shown in Table 2. Using the criteria outlined in the JBI tool [28]. Eight questions in total, Questions 3 and 4 on the JBI were not applicable and are excluded here. (1.Were the criteria for inclusion in the sample clearly defined? 2.Were the study subjects and the setting described in detail? 5.Were confounding factors identified? 6.Were strategies to deal with confounding factors stated? 7.Were the outcomes measured in a valid and reliable way? 8.Was appropriate statistical analysis used?). The sampling is clear. The information on participants is described in detail. All studies used objective and valid measures. While appropriate statistical analyses were conducted in all studies, fewer studies considered confounding factors or used strategies to deal with them. All studies reported Pearson correlation coefficients as outcomes. According to the quality assessment using the JBI tool, a total of 22 studies were assessed as medium quality, while other 11 studies were assessed as high quality. Overall, the quality of the included studies in this meta-analysis was good.

**Table 2:** Quality appraisal of cross-sectional surveys using the JBI tool.

Author	1.	2.	5.	6.	7.	8.	Quality
Neff et al. [34]	Yes	Yes	No	No	Yes	Yes	Medium
Hardin et al. [15]	Yes	Yes	No	No	Yes	Yes	Medium
Ogunyemi & Mabekoje [35]	Yes	Yes	No	No	Yes	Yes	Medium
Shorey et al. [36]	Yes	Yes	Yes	Yes	Yes	Yes	High
Oluyinka et al. [37]	Yes	Yes	Yes	Yes	Yes	Yes	High
Weigold & Robitschek [4]	Yes	Yes	No	No	Yes	Yes	Medium
Ayub & Iqbal [11]	Yes	Yes	No	No	Yes	Yes	Medium
Malik et al. [12]	Yes	Yes	Yes	Yes	Yes	Yes	High
Yakunina et al. [38]	Yes	Yes	Yes	Yes	Yes	Yes	High
Loo et al. [13]	Yes	Yes	No	No	Yes	Yes	Medium
Luyckx & Robitschek [39]	Yes	Yes	No	No	Yes	Yes	Medium
Çelik [10]	Yes	Yes	No	No	Yes	Yes	Medium
Beri & Jain [9]	Yes	Yes	No	No	Yes	Yes	Medium
Borowa et al. [40]	Yes	Yes	No	No	Yes	Yes	Medium
Yang & Chang [41]	Yes	Yes	No	No	Yes	Yes	Medium
Batool et al. [42]	Yes	Yes	Yes	Yes	Yes	Yes	High
Çankaya et al. [8]	Yes	Yes	Yes	Yes	Yes	Yes	High
Shigemoto et al. [43]	Yes	Yes	No	No	Yes	Yes	Medium
Hirata & Kamakura [44]	Yes	Yes	No	No	Yes	Yes	Medium
Kugbey et al. [45]	Yes	Yes	Yes	Yes	Yes	Yes	High
Aranha et al. [22]	Yes	Yes	No	No	Yes	Yes	Medium
Mason [46]	Yes	Yes	Yes	Yes	Yes	Yes	High
Arikkatt & Mohanan [47]	Yes	Yes	No	No	Yes	Yes	Medium
Noor et al. [48]	Yes	Yes	No	No	Yes	Yes	Medium
Cai & Lian [49]	Yes	Yes	Yes	Yes	Yes	Yes	High
Chowdhury et al. [50]	Yes	Yes	Yes	Yes	Yes	Yes	High
Hassan et al. [51]	Yes	Yes	No	No	Yes	Yes	Medium
Masood & Arshad [52]	Yes	Yes	No	No	Yes	Yes	Medium
Soylu et al. [53]	Yes	Yes	No	No	Yes	Yes	Medium
Weigold et al. [18]	Yes	Yes	Yes	Yes	Yes	Yes	High
Chen & Guo [54]	Yes	Yes	No	No	Yes	Yes	Medium
Green & Yıldırım [55]	Yes	Yes	No	No	Yes	Yes	Medium
Rahim et al. [56]	Yes	Yes	No	No	Yes	Yes	Medium

Note: Questions 3 and 4 on the JBI were not applicable and are excluded here.

### 4.4 Thematic findings

A total of 33 studies containing 14,740 college students from around the world were included in this meta-analysis. A total of 29 correlated factors related to the personal growth initiative among college students were extracted from the 33 included studies. In the present study, Pearson correlation coefficient (r) was used to calculate the effect size (Fisher's Z), for the same correlated factors from two or more studies will combine effect size (Fisher's Z), then report the summary Pearson correlation coefficient (Summary r), while correlated factors from only one study will be reported with the Pearson correlation coefficient (r) directly from that study. The Bonferroni-corrected P-value was calculated as 0.0017 (0.05/29), therefore, if the P-value less than 0.0019, the Pearson correlation coefficient (r) was considered statistically significant. The results of the analysis are shown in Table 3.

**Table 3:** Correlation between correlated factors and college students’ personal growth initiative.

Correlated Factors	K	N	I^2^	Effect Model	Z	95% CI	Egger	r	P
Well-Being [9,11,24,36,46,47,52]	7	2605	81.5%	Random-Effect Model	0.44	[0.35, 0.53]	0.975	0.41	0.000
Self-Efficacy [[8], [9], [10],^35^,^42^,^49^]	6	3542	96.5%	Random-Effect Model	0.53	[0.33, 0.73]	0.071	0.49	0.000
Self-Esteem [12,22,39,44,50]	5	1306	32.1%	Fixed-Effect Model	0.29	[0.23, 0.34]	0.929	0.28	0.000
Optimism [34,36,41,53]	4	3037	86.8%	Random-Effect Model	0.42	[0.29, 0.56]	0.547	0.40	0.000
Subjective Happiness [34,45,46,51]	4	1072	82.1%	Random-Effect Model	0.40	[0.26, 0.55]	0.333	0.38	0.000
Depression [39,41,43]	3	1070	35.7%	Fixed-Effect Model	−0.22	[-0.28, −0.16]	0.444	−0.21	0.000
Hope [13,36,41]	3	1406	96.8%	Random-Effect Model	0.57	[0.25, 0.89]	0.266	0.52	0.000
Life Satisfaction [41,48,55]	3	913	96.3%	Random-Effect Model	0.51	[0.16, 0.86]	0.096	0.47	0.004
Mindfulness [13,37,56]	3	1553	94.3%	Random-Effect Model	0.41	[0.19, 0.63]	0.650	0.39	0.000
Psychological Distress [11,15,36]	3	662	75.9%	Random-Effect Model	−0.25	[-0.42, −0.08]	0.979	−0.24	0.003
Self-Compassion [22,34,47]	3	643	87.0%	Random-Effect Model	0.56	[0.33, 0.78]	0.120	0.51	0.000
Social Support [10,40,49]	3	2285	94.9%	Random-Effect Model	0.39	[0.11, 0.67]	0.129	0.37	0.007
Anxiety [24,41]	2	705	87.1%	Random-Effect Model	−0.27	[-0.49, −0.05]	N/A	−0.26	0.017
Emotional Intelligence [45,51]	2	660	0.0%	Fixed-Effect Model	0.47	[0.40, 0.55]	N/A	0.44	0.000
Negative Affect [15,34]	2	311	0.0%	Fixed-Effect Model	−0.26	[-0.37, −0.15]	N/A	−0.25	0.000
Personal Control [4,54]	2	1261	92.3%	Random-Effect Model	0.53	[0.33, 0.74]	N/A	0.49	0.000
Positive Affect [15,34]	2	311	0.0%	Fixed-Effect Model	0.52	[0.41, 0.63]	N/A	0.48	0.000
Academic Achievement [12]	1	150	N/A	N/A	0.56	[0.40, 0.72]	N/A	0.51	0.000
Acculturative Stress [38]	1	386	N/A	N/A	−0.14	[-0.24, −0.04]	N/A	−0.14	0.006
Family Functioning [52]	1	198	N/A	N/A	0.28	[0.14, 0.42]	N/A	0.27	0.000
Fear of Negative Evaluation [15]	1	134	N/A	N/A	−0.19	[-0.36, −0.02]	N/A	−0.19	0.028
Mental Heath [35]	1	425	N/A	N/A	0.11	[0.02, 0.21]	N/A	0.11	0.023
Posttraumatic Stress [43]	1	292	N/A	N/A	−0.25	[-0.36, −0.13]	N/A	−0.24	0.000
Problem-Solving Confidence [4]	1	478	N/A	N/A	0.50	[0.41, 0.59]	N/A	0.46	0.000

Correlated Factors	K	N	I^2^	Effect Model	Z	95% CI	Egger	r	P
Psychological Adjustment [38]	1	386	N/A	N/A	0.40	[0.30, 0.50]	N/A	0.38	0.000
Risk-Taking [35]	1	425	N/A	N/A	0.27	[0.17, 0.36]	N/A	0.26	0.000
Self-Determination [18]	1	818	N/A	N/A	0.31	[0.24, 0.38]	N/A	0.30	0.000
Sense of Purpose [49]	1	1912	N/A	N/A	0.93	[0.88, 0.97]	N/A	0.73	0.000
Smartphone Addiction [54]	1	783	N/A	N/A	0.35	[0.42, 0.28]	N/A	−0.34	0.000

Notes: K = Number of Studies; N = Number of participants; Z = Fisher's Z; r = Summary r; Egger = For Egger's test, the overall effect size (Fisher's Z) needs to be synthesized from at least three effect sizes, meaning that at least three studies need to be involved; P < 0.0017 (0.05/29) was statistically significant.

#### 4.4.1 Positive correlated factors

Before using the Bonferroni correction, 21 of the 29 correlated factors found were positively correlated with college students' personal growth initiative. After the Bonferroni correction, the results have shown that 18 factors significantly positively correlated with college students' personal growth initiative, including Well-Being (r = 0.41, P < 0.0017); Self-Efficacy (r = 0.49, P < 0.0017); Self-Esteem (r = 0.28, P < 0.0017); Optimism (r = 0.40, P < 0.0017); Subjective Happiness (r = 0.38, P < 0.0017); Hope (r = 0.52, P < 0.0017); Mindfulness (r = 0.39, P < 0.0017); Self-Compassion (r = 0.51, P < 0.0017); Emotional Intelligence (r = 0.44, P < 0.0017); Personal Control (r = 0.49, P < 0.0017); Positive Affect (r = 0.48, P < 0.0017); Academic Achievement (r = 0.51, P < 0.0017); Family Functioning (r = 0.27, P < 0.0017); Problem-Solving Confidence (r = 0.46, P < 0.0017); Psychological Adjustment (r = 0.38, P < 0.0017); Risk-Taking (r = 0.26, P < 0.0017); Self-Determination (r = 0.30, P < 0.0017); Sense of Purpose (r = 0.73, P < 0.0017). While the other three including Life Satisfaction (r = 0.47, P > 0.0017); Social Support (r = 0.37, P > 0.0017); Mental Health (r = 0.11, P > 0.0017) were not statistically significantly positively correlated with college students' personal growth initiative after the Bonferroni correction. Among the 18 positively correlated factors, 7 correlated factors were involved in one study each, while the other 11 had two or more studies, and all the correlations (r values) between correlated factors and college students’ personal growth initiative were supported by larger sample sizes.

#### 4.4.2 Negative correlated factors

Before using the Bonferroni correction, 8 of the 29 correlated factors found were negatively correlated with college students' personal growth initiative. After the Bonferroni correction, the results have shown that 4 factors significantly negatively correlated with college students' personal growth initiative, including Depression (r = −0.21, P < 0.0017); Negative Affect (r = −0.25, P < 0.0017); Posttraumatic Stress (r = −0.24, P < 0.0017); Smartphone Addiction (r = −0.34, P < 0.0017). However, Psychological Distress (r = −0.24, P > 0.0017); Anxiety (r = −0.26, P > 0.0017); Acculturative Stress (r = −0.14, P > 0.0017); Fear of Negative Evaluation (r = −0.19, P > 0.0017) was not statistically significantly negatively correlated with college students' personal growth initiative after the Bonferroni correction. Among the 4 significant negatively correlated factors, 2 negatively correlated factors were involved in one study each, while the other 2 negatively correlated factors involved two or more studies, and all the correlations (r values) between correlated factors and college students’ personal growth initiative were supported by larger sample sizes.

### 4.5 Sensitivity and publication bias

For sensitivity, the correlated factors were compared using the change analysis model, and the Fisher's Z values were still found to be statistically significant, indicating that the outcome was stable. Egger's test was used to determine whether there was publication bias (due to the Egger's test requires a minimum of three effect sizes to be combined, meaning that the correlated factor must involve at least three studies to undergo Egger's test), and the results of Egger's test showed that all P values were >0.05 (Table 3), so no publication bias in this study.

## 5 Discussion

In this international literature review, a total of 29 factors were found to be correlated with college students' personal growth initiative by screening and extracting the included literature, of which 21 were positively correlated and 8 were negatively correlated. After Bonferroni correction, 18 factors such as happiness, self-efficacy and self-esteem had statistically significant positively correlated with college students' personal growth initiative, and 4 factors such as depression, and negative emotions had statistically significant negatively correlated with college students’ personal growth initiative.

### 5.1 Factors positively correlated with college students’ personal growth initiative

After Bonferroni correction, there were 18 factors that were statistically significantly and positively correlated with college students’ personal growth initiatives. Based on the Pearson correlation coefficient level criteria, the 18 significantly positive correlation factors were classified into 4 high-level positively correlated factors, 10 medium-level positively correlated factors and 4 low-level positively correlated factors.

#### 5.1.1 High-level positively correlated factors

High-level positively correlated factors of college students' personal growth initiatives include Sense of Purpose (r = 0.73, P < 0.0017), Hope (r = 0.52, P < 0.0017), Self-Compassion (r = 0.51, P < 0.0017), and Academic Achievement (r = 0.51, P < 0.0017). Active participation in personal growth activities can help students to discover and develop their potential and thus realize their aspirations for self-fulfilment [57], which is inextricably associated with having the clear sense of purpose and hope for the future. Having a clear sense of purpose motivates and provides direction to college students in their pursuit of growth and progress [49,58], while having positive hope in the future helps college students to actively engage in personal growth activities and set high standards for their own development [59]. This suggests that college students need to have a clear understanding of their sense of purpose and have positive future hopes that will propel them to progress in their personal growth. While college students who accept themselves, with more self-compassion are better able to overcome difficulties and achieve personal growth [34,60], suggest that self-compassion provides them with the inner support and motivation to overcome challenges and succeed. In addition, academic achievement reflects to a large extent a student's learning attitude and effort, students who study hard will tend to achieve better academic results [61]. This attitude to learning is in itself an expression of personal growth initiative, as they proactively pursue knowledge and learning opportunities, thereby contributing to their own growth and development [62].

#### 5.1.2 Medium-level positively correlated factors

Medium-level positively correlated factors of college students' personal growth initiatives include Well-Being (r = 0.41, P < 0.0017); Self-Efficacy (r = 0.49, P < 0.0017); Optimism (r = 0.40, P < 0.0017); Subjective Happiness (r = 0.38, P < 0.0017); Mindfulness (r = 0.39, P < 0.0017); Emotional Intelligence (r = 0.44, P < 0.0017); Personal Control (r = 0.49, P < 0.0017); Positive Affect (r = 0.48, P < 0.0017); Problem-Solving Confidence (r = 0.46, P < 0.0017); Psychological Adjustment (r = 0.38, P < 0.0017). Firstly, well-being and subjective happiness represent college students' perceptions and subjective feelings about their own life [63,64], reflect college student's degree of satisfaction with life and positive emotions, and are critical in shaping a positive mindset in personal growth. Secondly, self-efficacy, personal control and problem-solving confidence indicate college student's confidence [65,66] and trust in their own abilities and control. These factors are important for individuals to overcome challenges, solve problems and achieve personal growth, and contribute to the development of self-confidence and coping skills. Thirdly, optimism, positive affect and mindfulness point to college student's positive outlook on the future and focus on and acceptance of the present [67,68]. These factors help college students to maintain a positive and optimistic attitude in the face of challenges and stress, and promote personal growth and development. Finally, emotional intelligence and psychological adjustment emphasize college student's ability to recognize and regulate emotions [69]. This is essential for college students to cope with the various emotions and stresses in their lives, and helps them to better adapt to changes in their environment and achieve personal growth.

#### 5.1.3 Low-level positively correlated factors

Low-level positively correlated factors of college students' personal growth initiatives include Self-Esteem (r = 0.28, P < 0.0017), Family Functioning (r = 0.27, P < 0.0017), Risk-Taking (r = 0.26, P < 0.0017), and Self-Determination (r = 0.30, P < 0.0017). Firstly, the establishment and maintenance of self-esteem is crucial for developing a healthy self-concept and a positive mindset [70]. By having good self-esteem, college students are more able to cope with challenges and maintain a positive and optimistic attitude [71], thus promoting their personal growth and development. Secondly, the family plays a crucial role in college students’ growth process [72], and good family functioning implies good communication, support and understanding among family members, and this family environment provides college students with stable emotional support and a positive emotional atmosphere so that they can feel safe and inspired in the face of challenges and difficulties [52,73]. Thirdly, in the face of new challenges, college students need to overcome fear and uncertainty, which helps foster their self-growth and development. While moderate level of risk-taking helps college students to expand their horizons, increase their experience, and grow with more opportunities and experiences to overcome fears and challenges, and to develop personal courage and self-confidence [35]. Finally, self-determination refers to the autonomy and self-control that college students demonstrate in deciding the direction, goals and behavior of their lives [74]. This ability enables college students to better manage their lives and thus achieve personal growth and development. Although these factors significant correlation with personal growth initiative is low, they are still some correlated with the personal growth of college students should be noted.

### 5.2 Factors negatively correlated with college students’ personal growth initiative

After Bonferroni correction, there were 4 factors that were statistically significantly and negatively correlated with college students’ personal growth initiatives. Based on the Pearson correlation coefficient level criteria, the 4 significantly positive correlation factors were classified into 1 medium-level negatively correlated factors and 3 low-level negatively correlated factors.

#### 5.2.1 Medium-level negatively correlated factors

Only Smartphone Addiction (r = −0.34, P < 0.0017) was medium-level negative correlated with college students' personal growth initiative. With the popularity and convenience of smartphones, more and more college students may fall into the quagmire of smartphone addiction [75,76], and this addictive behavior may become a major obstacle restricting their personal growth. Smartphone addiction may cause college students to become overly addicted to using their smartphones and neglect opportunities for personal growth [54,77], as well as addiction to smartphone may reduce college students' academic engagement, social interactions, and life satisfaction [78], taking them away from a positive growth trajectory. Moreover, the moderate negative correlation between smartphone addiction and college students' personal growth initiative seems to suggest that various types of addictive behaviors may be potential negative correlated factors of college students’ personal growth initiative.

#### 5.2.2 Low-level negatively correlated factors

Low-level negatively correlated factors of college students' personal growth initiatives include Depression (r = −0.21, P < 0.0017); Negative Affect (r = −0.25, P < 0.0017); Posttraumatic Stress (r = −0.24, P < 0.0017). The presence of these low negative correlations also suggests the reality of the challenges and pressures that college students face in their personal growth process, their existence is important to our comprehension of the importance of personal growth among college students. On the one hand, mental health problems such as depression, negative affect and posttraumatic stress showed negative correlations with personal growth initiatives among college students, suggesting that poor mental health status may hinder college students’ positive personal growth and self-development [24]. However, on the other hand, it also suggests that having good personal growth initiatives may enable university students to cope with these adverse situations effectively [11,79].

### 5.3 Limitations and future directions

To the best of the author's knowledge, the present study is the most systematic and comprehensive meta-analysis report to date exploring the factors correlated with college students' personal growth initiative. Using a rigorous and scientific approach, the combination of 33 studies involving 14,740 college students provides an in-depth understanding of all the factors correlated with personal growth initiatives among college students explored in existing research. In addition, the quality of the included studies was good, and the results of this study were stable and without publication bias, making the results relatively reliable. However, there are some limitations to the findings of this study as follows.

Firstly, this study used the results of heterogeneity tests as the basis for selecting the effects model. However, due to the limited number of studies for each correlated factor, this study was unable to conduct subgroup analyses or regression analyses to explore heterogeneity for those correlated factors where the random-effect model was chosen. Secondly, most of the studies included in this meta-analysis were from countries in the Americas, Asia and Africa, with few from Europe, and none from Oceania. This led to the fact that although the study included a large sample sizes, there may still be a bias in the source of the sample, affecting the general applicable of the findings. The absence of research in other regions is not just a limitation of this study but a significant research gap in the field. Finally, this study explored the factors correlated with college students' personal growth initiative based on correlation, so it was unable to determine the causal relationship between these factors and college students’ personal growth initiative.

To fill the limitations of this study, firstly, future research may be able to include more studies when the field of research on personal growth initiatives among college students is better developed, which would then allow for an exploration of the heterogeneity of findings. Secondly, research related to college students' personal growth initiatives in underrepresented regions can not only validate the general applicable of this study's findings, but also fill in the gaps in this current field of research. Finally, this study suggests that future research should focus on causality and conduct research using longitudinal design or experimental design to reveal the causal relationship between these correlated factors and college students' personal growth initiative.

## 6 Conclusion

This study conducted a comprehensive quantitative review of existing research on the factors correlated with college students' personal growth initiative, and identified 18 factors that are positively correlated and 4 factors that are negatively correlated with college students' personal growth initiative, as well as the level of correlation. These findings provide important information for understanding personal growth initiative among college students, and provide reference for educators to develop individualized interventions targeted at improving college students’ personal growth initiatives.

## Funding statement

This research did not receive any specific grant from funding.

## Data availability statement

The data use in this study have not been deposited into a publicly available repository. Data will be made available on request.

## Ethics statement

Not applicable.

## CRediT authorship contribution statement

**Zun Jiao:** Writing – original draft, Validation, Software, Methodology, Data curation. **You Chen:** Validation, Software, Data curation. **Chunwei Lyu:** Writing – review & editing, Writing – original draft, Supervision, Methodology, Conceptualization.

## Declaration of competing interest

The authors declare that they have no known competing financial interests or personal relationships that could have appeared to influence the work reported in this paper.
